# 

**Published:** 1890-03

**Authors:** 


### THE BRISTOL

### Medico-Chirurgical Journal.

A JOURNAL OF THE MEDICAL SCIENCES FOR THE WEST OF ENGLAND AND SOUTH WALES

*PUBLISHED UNDER THE AUSPICES OF*

*THE BRISTOL MEDICO-CHIRURGICAL SOCIETY.*

EDITOR:

J. GREIG SMITH, M.A., F.R.S.E.

ASSISTANT-EDITOR:

L. M. GRIFFITHS.

"Scire est nescire, nisi id me Scire alius sciret."

*VOL. VIII.*

BRISTOL: J. W. ARROWSMITH;

LONDON: J. & A. CHURCHILL, 11 NEW BURLINGTON STREET.

1890.

L. H.

R31
.B8

### CONTENTS OF VOLUME VIII.

ORIGINAL PAPERS: *Page*

Personal Methods employed in the Radical Cure of Hernia—Inguinal, Femoral and Umbilical. By J. GREIG SMITH ... 1

The Sequelæ of Suppurative Otitis Media. By F. H. EDGEWORTH, B.A., M.B., B.Sc.... ... ... ... ... ... ... 18, 87

Post-partum Complications. By A. E. AUST LAWRENCE, M.D. 73

The Symptoms and Treatment of Outgrowths from the Back of the Tongue. By BARCLAY J. BARON, M.B. ... ... ... 87

On Some Points in the Surgical Physiology of the Abdomen and Pelvis. By T. S. ELLIS ... ... ... ... ... ... ... 98

Epidemic Influenza. By WM. JOHNSTONE FYFFE, M.D. ... ... 147

The Bristol Infirmary in my Student Days, 1822—1828. By HENRY ALFORD, F.R.C.S. ... ... ... ... ... ... ... ... 165

Multiple Cancellous Exostoses. By EWEN J. MACLEAN, M.B., C.M. ... ... ... ... ... ... ... ... ... ... ... ... ... 217

On Some Varieties of Paraplegia with Lateral Sclerosis, with Cases. By J. MICHELL CLARKE, M.A., M.B., M.R.C.P. ... 226

CLINICAL RECORDS:

A Case of Factitious Urticaria. By HENRY WALDO, M.D., M.R.C.P. ... ... ... ... ... ... ... ... ... ... ... ... 29

Factitious Urticaria. By ALFRED J. HARRISON, M.B. ... .. 32

A Case of Acute Ascending Paralysis (Landry's Paralysis). By ARTHUR G. BLOMFIELD, M.D. .. ... ... ... ... ... ... 113

Page

Case of "Orbital Aneurism" Treated by Ligature of the Carotid. By A. W. Prichard, M.R.C.S. ... ... ... ... ... ... ... 115

Case of Epithelioma of the Body of the Uterus. By J. G. Swayne, M.D.... ... ... ... ... ... ... ... ... ... ... 119

Clinical Studies of Disease in Children. By Eliza L. Walker Dunbar, M.D., L.K.Q.C.P.I. ... ... ... ... ... ... ... 162

A Case of Congenital Heart-Disease. By Lockhart Stephens, M.R.C.S., L.S.A. ... ... ... ... ... ... ... ... ... ... 196

Some Cases of Peripheral Neuritis. By D. R. Paterson, M.D., M.R.C.P. ... ... ... ... ... ... ... ... ... ... ... ... 244

Health-Resorts in the West of England and South Wales: Ilfracombe. By E. J. Slade-King, M.D., D.P.H. ... ... ... 257

Reviews of Books ... ... ... ... ... ... ... 36, 126, 199, 268

Notes on Preparations for the Sick ... ... ... ... 46, 134, 206, 275

The International Medical Congress ... ... ... ... ... ... 49

Periscope ... ... ... ... ... ... ... ... ... ... ... 54, 137, 211, 278

Scraps ... ... ... ... ... ... ... ... ... ... ... 71, 145, 215, 287

### Bristol Medico-Chirurgical Society.

### President.
NELSON C. DOBSON.

### President-Elect.

S. H. SWAYNE.

### Honorary Secretary.
G. MUNRO SMITH.

### Committee.

*Past Presidents.*
R. W. COE.
AUGUSTIN PRICHARD, M.D. Berlin.
J. G. SWAYNE, M.D. Lond.
E. LONG FOX, M.D. Oxon.
JAMES G. DAVEY, M.D. St. And.
W. JOHNSTONE FYFFE, M.D. Dub.
G. F. BURDER, M.D. Aberd.
W. H. SPENCER, M.D., M.A. Cantab.
F. POOLE LANSDOWN.
L. M. GRIFFITHS.
R. SHINGLETON SMITH, M.D., B.Sc. Lond.

F. RICHARDSON CROSS, M.B. Lond.
A. J. HARRISON, M.B. Lond.
W. H. HARSANT.
J. E. SHAW, M.B., C.M. Ed.
E. MARKHAM SKERRITT, M.D., B.S., B.A. Lond.
J. GREIG SMITH, M.B., C.M., M.A. Aberd., F.R.S.E.

Medical Men wishing to join the Society are requested to communicate with the Honorary Secretary, G. Munro Smith, 27 All Saints' Road, Clifton, Bristol. The Annual Subscription is 10/6.

---

*Extracts from Journal By-laws.*

4.—The *Journal* shall be delivered free to Subscribers and also to Members of the Society whose subscriptions are not in arrear.

6.—The Annual Subscription to the *Journal* for those who pay before the 1st of July shall be 5/-, post free, or 6/- if paid after that date.

---

Communications intended for insertion in the *Journal* should be sent to the Editor, J. Greig Smith, M.A., F.R.S.E., 16 Victoria Square, Clifton, Bristol.

Books for Review, Exchange Journals, and communications referring to the delivery of the *Journal* should be sent to the Assistant-Editor, L. M. Griffiths, 9 Gordon Road, Clifton, Bristol, to whom Subscriptions are to be paid *in advance*.

Authors requiring Reprints of their Papers should communicate with the Publisher.

Cases for Binding Vols. I.–VII. are now ready, and may be obtained, price 7*d*. each (postage 2*d*. extra), upon application to J. W. Arrowsmith, Quay Street, Bristol; or the *Journal* will be bound, including Case, for 1/3 per volume.

### Bristol Medico-Chirurgical Society.

---

### PAST PRESIDENTS.

1874—75. FREDERICK BRITTAN, M.D., B.A. Dub.

1875—76. ROBERT WILLIAM COE.

1876—77. SAMUEL MARTYN, M.D. Ed.

1877—78. AUGUSTIN PRICHARD, M.D. Berlin.

1878—79. HENRY EDWARD FRIPP, M.D. Würzburg.

1879—80. WILLIAM MICHELL CLARKE.

1880—81. JOSEPH GRIFFITHS SWAYNE, M.D. Lond.

1881—82. EDWARD LONG FOX, M.D. Oxon.

1882—83. JAMES GEORGE DAVEY, M.D. St. And.

1883—84. WILLIAM JOHNSTONE FYFFE, M.D. Dub.

1884—85. GEORGE FORSTER BURDER, M.D. Aberd.

1885—86. WILLIAM HENRY SPENCER, M.D., M.A. Cantab.

1886—87. FRANCIS POOLE LANSDOWN.

1887—88. LEMUEL MATTHEWS GRIFFITHS.

1888—89. ROBERT SHINGLETON SMITH, M.D., B.Sc. Lond.

1890.

### LIST OF SUBSCRIBERS

TO THE

### Bristol Medico-Chirurgical Journal.

*Those marked * are Members of the Society.*

| *Name.* | *Residence.* |
|---|---|
| ABERCROMBIE, JOHN, M.D. Cantab. ... ... ... | 23 Upper Wimpole Street, London, W. |
| ACKLAND, J. MACKNO ... ... ... ... ... ... | 24 Southernhay, Exeter. |
| *ACKLAND, W. R. ... ... ... ... ... ... ... | 5 Rodney Place, Clifton. |
| ADAMS, GEORGE ... ... ... ... ... ... ... | 11 Westbourne Place, Clifton. |
| *ADAMS, JOHN BARFIELD ... ... ... ... ... ... | 110 Cotham Road, Bristol. |
| ALEXANDER, JAMES, M.D., M.Ch., B.A. Dub. | Bishop's Place, Paignton, Devonshire. |
| *ALFORD, GEORGE ERNEST ... ... ... ... ... | 5 Royal Crescent, Weston-super-Mare |
| ALFORD, HENRY J., M.D. Lond. ... ... ... | 2 Marlborough Terrace, Taunton. |
| ANSTIE, THOMAS B. ... ... ... ... ... ... | 21 Northgate Street, Devizes. |
| APPLETON, HENRY, M.D. Aberd. ... ... ... | Poulton Lodge, Stoke Bishop. |
| ARTHUR, JOHN ... ... ... ... ... ... ... ... | Hilton, Llandaff. |
| *ATCHLEY, GEORGE F., M.B. Lond. ... ... ... | Tyndall's Park, Bristol. |
| *AVELINE, H. T. S. ... ... ... ... ... ... | Bailbrook House, Bath. |
| | |
| BAKER, A. de W. ... ... ... ... ... ... ... | 2 Lawn Terrace, Dawlish. |
| *BARCLAY, W. M. ... ... ... ... ... ... ... | Queen's Road, Clifton. |
| BARETTI, T. G. L'ENARDI ... ... ... ... ... | 49 Royal York Crescent, Clifton. |
| *BARON, BARCLAY J., M.B., C.M. Ed. ... ... | 16 White Ladies Road, Clifton. |
| BARTLETT, B. POPE ... ... ... ... ... ... | Bourton, Dorsetshire. |

### LIST OF SUBSCRIBERS.

| | |
|---|---|
| *Basset, Walter ... ... ... ... ... ... ... | 24 Stow Hill, Newport, Mon. |
| Batten, Rayner W., M.D. Lond. ... ... ... | 1 Brunswick Square, Gloucester. |
| Batten, W. Smith ... ... ... ... ... ... | Curzon House, Calne. |
| Beale, Lionel S., M.B. Lond., F.R.S. ... | 61 Grosvenor Street, London, W. |
| Beatson, W. B., M.D. St. And. ... ... ... | Grange Road, Eastbourne. |
| *Beddoe, John, M.D. Ed., B.A. Lond., F.R.S. | The Manor House, Clifton. |
| Benham, Harry A., M.D., C.M. Aberd. ... | Asylum, Stapleton, Gloucestershire. |
| Bennett, W. S. ... ... ... ... ... ... ... | Escot, Penzance. |
| *Benson, A. H. ... ... ... ... ... ... ... | Wrington. |
| Berry, F. C., M.D., B.Ch., B.A. Dub. ... ... | Lyn Cottage, Lynton. |
| Bevan, T. W. ... ... ... ... ... ... ... ... | Nantyglo, Monmouthshire. |
| Bisdée, Alfred J.... ... ... ... ... ... ... | Weston-super-Mare. |
| *Blacker, A. E. ... ... ... ... ... ... ... | Richmond Hill Avenue, Clifton. |
| Blacker, E. ... ... ... ... ... ... ... ... | Midsomer Norton. |
| *Blagg, Arthur F. ... ... ... ... ... ... | 6 West Mall, Clifton. |
| Blomfield, Arthur G., M.D. Aberd. ... ... | 34 West Southernhay, Exeter. |
| *Board, Edmund C. ... ... ... ... ... ... | 11 Caledonia Place, Clifton. |
| Boys, Arthur Henry... ... ... ... ... ... | Chequer Street, St. Alban's. |
| Brabazon, A. B., M.D. Aberd. ... ... ... ... | 12 Darlington Street, Bath. |
| Brettingham, C. E. S. ... ... ... ... ... | Manor House, Stogumber. |
| Bright, J. A. ... ... ... ... ... ... ... ... | Glastonbury. |
| Broom, John, M.D., Ch.D., Obst.D. Brussels | St. Paul's Road, Clifton. |
| Brown, G. Arthur ... ... ... ... ... ... | Tredegar. |
| Brown, William, M.D., C.M. Glasg. ... ... | Fishponds, Gloucestershire. |
| Browne, A. E. Newbury ... ... ... ... ... | Burbage, Wiltshire. |
| Browne, John, M.D. Ed. ... ... ... ... ... | Dundalk. |
| Browne, G. Henry ... ... ... ... ... ... | The Hermitage, Brynmawr, Breconshire. |
| Browne, J. Walton, M.D., B.A. Qu. Univ. I. | 10 College Square North, Belfast. |
| Browne, Lennox ... ... ... ... ... ... ... | 36 Weymouth Street, London, W. |
| *Burder, George F., M.D. Aberd. ... ... ... | 63 Oakfield Road, Clifton. |
| Burges, R. E., M.D., M.Ch., B.A. Qu. Univ. I. | 3 Egerton Terrace, Hoole, Chester. |
| Burroughs, E. F. H. ... ... ... ... ... ... | 64 Bristol Road, Weston-super-Mare. |
| *Bush, J. Paul ... ... ... ... ... ... ... ... | 13 Lansdown Place, Clifton. |
| Buzzard, Thomas, M.D. Lond. ... ... ... | 74 Grosvenor Road, London, W. |

| | |
|---|---|
| *Calder, F. ... ... ... ... ... ... ... ... | 14 Belle Vue, Cotham, Bristol. |
| *Cameron, John, M.B., C.M. Glasg. ... ... | 63 Cotham Road, Bristol. |
| Cann, Francis M. ... ... ... ... ... ... ... | Sefton House, Dawlish. |
| Carey, J., M.D. Lond. ... ... ... ... ... ... | 2 Hamdon Villas, Taunton. |
| Carlyon, Thomas B. ... ... ... ... ... ... | Redruth. |
| *Carr, Alexander ... ... ... ... ... ... ... | Wells Road, Bristol. |
| Carter, R. W., M.D. Calcutta ... ... ... ... | 1 Bank Buildings, Weymouth. |
| *Carter, W. F.... ... ... ... ... ... ... ... | 48 Coronation Road, Bristol. |
| Cave, Edward J., M.D. Lond. ... ... ... ... | Crewkerne. |
| Clapp, W. J. ... ... ... ... ... ... ... ... | Lord Street, Penarth. |
| Clark, Thomas ... ... ... ... ... ... ... | Blair, Minehead, Somersetshire. |
| Clarke, Arthur, B.A. Lond. ... ... ... ... | Street, Somersetshire. |
| *Clarke, John Michell, M.B., M.A. Cantab. | Clifton Road, Clifton. |
| Clay, Challoner ... ... ... ... ... ... ... | Manor House, Fovant. |
| Clay, R. Hogarth, M.D. Ed. ... ... ... ... | 4 Windsor Villas, Plymouth. |
| Coates, Charles ... ... ... ... ... ... ... | 10 Circus, Bath. |
| Coates, Harcourt ... ... ... ... ... ... | 17 Endless Street, Salisbury. |

### LIST OF SUBSCRIBERS.

| | |
|---|---|
| Coathupe, Edwin W. ... ... ... ... ... ... | 7 Clifton Park, Clifton. |
| *Coe, R. W. ... ... ... ... ... ... ... ... | 7 Pembroke Road, Clifton. |
| Coker, T. V. ... ... ... ... ... ... ... ... | 2 Chesterfield Place, Clifton. |
| Cole, Thomas, M.D. Lond. ... ... ... ... | 18 Circus, Bath. |
| *Collins, H. W. ... ... ... ... ... ... ... ... | Wrington, Somersetshire. |
| Collyns, Robert J. ... ... ... ... ... ... | Dulverton, Somersetshire. |
| Colman, Thomas J., M.D. Ed.... ... ... ... | Elmgrove Road, Bristol. |
| Colmer, Ptolemy S. H., M.D. Dur. ... ... | South Street House, Yeovil. |
| Cooke, A. S. ... ... ... ... ... ... ... ... | Badbrook House, Stroud. |
| *Corbould, George G. ... ... ... ... ... | 22 Berkeley Square, Bristol. |
| *Cotton, William, M.B., C.M. Ed. ... ... | 227 Gloucester Road, Bristol. |
| Cousins, J. Ward, M.D. Lond. ... ... ... | Kent Road, Southsea. |
| Cowan, F. S. ... ... ... ... ... ... ... ... | 27 Queen Square, Bath. |
| Cox, William I. ... ... ... ... ... ... ... | Hawkesbury-Upton. |
| Craddock, Samuel ... ... ... ... ... ... | 1 Green Park, Bath. |
| Crespi, Alfred J. H. ... ... ... ... ... | Poole Road, Wimborne. |
| *Cross, F. Richardson, M.B. Lond. ... ... | Richmond Park Road, Clifton. |
| *Crossman, Edward, M.D. Dur. ... ... ... | Hambrook, Gloucestershire. |

| | |
|---|---|
| *Dacre, John ... ... ... ... ... ... ... ... | St. Paul's Road, Clifton. |
| Daniel, T. P. ... ... ... ... ... ... ... ... ... | Beaminster, Dorsetshire. |
| *Davey, James G., M.D. St. And. ... ... ... | Thistledene, Surbiton. |
| *Davies, D. S., M.B. Lond. ... ... ... ... | 60 Oakfield Road, Clifton. |
| Davies, E. Cluneglas ... ... ... ... ... | Pontfaen, Lampeter, Cardiganshire. |
| Davies, H. N. ... ... ... ... ... ... ... ... ... | Porth, Glamorganshire. |
| Davis, Robert ... ... ... ... ... ... ... ... | 14 St. James's Square, Bath. |
| Davis, Theodore, M.D. Lond. ... ... ... | Beachcroft, Clevedon. |
| Day, Percy Howard ... ... ... ... ... ... | Stalmine, Poulton-le-Fylde. |
| Deas, P. Maury, M.B., M.S. Lond. ... ... | Wonford House, Exeter. |
| Dening, Edwin ... ... ... ... ... ... ... ... | Stow-on-the-Wold. |
| Devis, Harry F. ... ... ... ... ... ... ... ... | Wells Road, Bristol. |
| Devonald, Alfred ... ... ... ... ... ... | Rhoscelyn House, Cowbridge. |
| *Dew, Henry R. ... ... ... ... ... ... ... ... | 25 Berkeley Square, Bristol. |
| *Dobson, Nelson C. ... ... ... ... ... ... | 27 Victoria Square, Clifton. |
| *Dowson, W., M.B., B.C., M.A. Cantab. ... ... | Great George Street, Bristol. |
| Doyne, Robert W. ... ... ... ... ... ... | Southbourne, Oxford. |
| Duffett, E. D. ... ... ... ... ... ... ... ... | 5 London Street, Swindon, Wiltshire. |
| Dunbar, Eliza L. Walker, M.D. Zürich ... | 9 Oakfield Road, Clifton. |
| *Duncan, William ... ... ... ... ... ... ... | 17 Redland Grove, Bristol. |

| | |
|---|---|
| Eadon, W. F. Bailey ... ... ... ... ... ... | Hambrook, Gloucestershire. |
| *Eager, Reginald, M.D. Lond. ... ... ... | Northwoods, Winterbourne. |
| Eddowes, Charles ... ... ... ... ... ... | Maddington, Devizes. |
| Edgeworth, T. F.... ... ... ... ... ... ... | 11 Victoria Road, Cotham, Bristol. |
| Edmunds, R. ... ... ... ... ... ... ... ... | Pontnewydd. |
| Edwardes, Thomas ... ... ... ... ... ... | Llansaintffraid, Montgomeryshire |
| Edwards, W. T., M.D. Lond. ... ... ... ... | Springfield House, Cardiff. |
| Elder, William ... ... ... ... ... ... ... ... | 3 Trelawney Rood, Bristol. |
| *Elliott, Christopher, M.D., B.A. Dub. ... | 88 Pembroke Road, Clifton. |

### LIST OF SUBSCRIBERS.

Ellis, Henry V., M.B., C.M. Aberd. ... ... Ty Bryn, Reynoldstone.

Ellis, T. S. ... ... ... ... ... ... ... ... 6 Clarence Street, Gloucester.

Ellis, W. Mc.D., M.D. Brussels ... ... ... 7 Alexander Buildings, Bath.

Embleton, Dennis C. ... ... ... ... ... ... St. Michael's Road, Bournemouth.

*Etches, W. R. ... ... ... ... ... ... ... 13 Dowry Square, Clifton.

Evans, J. Fenton, M.B., C.M. Ed. ... ... 6 Whitehall Yard, London, S.W.

Evans, W. G. ... ... ... ... ... ... ... ... Beckington.

*Ewens, John ... ... ... ... ... ... ... ... 14 White Ladies Road, Clifton.

Fairbanks, W., M.D., C.M. Ed. ... ... ... Wells, Somersetshire.

*Fendick, R. George ... ... ... ... ... ... St. Paul's Road, Clifton.

Fernie, James ... ... ... ... ... ... ... ... Stratton St. Margaret.

Finch, Thomas, M.D. St. And. ... ... ... Westville, St. Mary Church.

Fisher, Fred. B. ... ... ... ... ... ... ... West Walks, Dorchester.

Flemming, C. E. S. ... ... ... ... ... ... Freshford.

Ford, Joseph ... ... ... ... ... ... ... ... Wedmore.

Forty, D. H. ... ... ... ... ... ... ... ... Wotton-under-Edge.

Fox, Arthur E. W., M.B., C.M. Ed. ... ... 16 Gay Street, Bath.

*Fox, Bonville B., M.D., M.A. Oxon. ... ... Brislington.

Fox, Charles Henry, M.D. St. And. ... ... Brislington.

*Fox, E. Long, M.D. Oxon. ... ... ... ... Church House, Clifton.

Fox, John Henry ... ... ... ... ... ... ... Tregea House, Penzance.

Fraser, D. A., M.D. Brussels ... ... ... ... Pomeroy House, Totnes.

Fraser, Græme B. ... ... ... ... ... ... Beach Road, Weston-super-Mare.

Freeman, Henry W. ... ... ... ... ... ... 24 Circus, Bath.

Fry, John B. ... ... ... ... ... ... ... ... Ashley Lodge, Esher.

Furse, Edwin ... ... ... ... ... ... ... ... South Molton.

*Fyffe, W. Johnstone, M.D., B.A. Dub. ... 2 Rodney Place, Clifton.

Gamble, C. H.... ... ... ... ... ... ... ... Barnstaple.

Gardiner, George ... ... ... ... ... ... ... 1 Redcliff Hill, Bristol.

*Gibbs, Alfred N. Godby ... ... ... ... ... 27 Chandos Road, Bristol.

Gill, John, M.D. Brussels... ... ... ... ... 31 Apsley Road, Clifton.

*Gloag, G. A. ... ... ... ... ... ... ... ... 7 College Green, Bristol.

Goodden, Wyndham C. ... ... ... ... ... 86 Stapleton Road, Bristol.

Goodridge, Henry F. A., M.D. Lond. ... 10 Brock Street, Bath.

Goss, T. Biddulph ... ... ... ... ... ... 1 Circus, Bath.

Gould, A. Pearce, M.B., M.S. Lond. ... ... 10 Queen Anne Street, London, W.

Grace, Alfred ... ... ... ... ... ... ... ... Chipping Sodbury.

Grace, Edward Mills ... ... ... ... ... Thornbury, Gloucestershire.

Grace, Henry ... ... ... ... ... ... ... ... Kingswood, Bristol.

Grace, W. G. ... ... ... ... ... ... ... ... 57 Stapleton Road, Bristol.

Gray, F. A. ... ... ... ... ... ... ... ... Ottery St. Mary.

Green, F. K. ... ... ... ... ... ... ... ... 3 Gay Street, Bath.

Gregory, George S. ... ... ... ... ... ... Southampton Row, London.

*Griffiths, L. M. ... ... ... ... ... ... ... 9 Gordon Road, Clifton.

Grigor, Macadam ... ... ... ... ... ... ... Alexandra Avenue, London, S.W.

Grote, G. W., M.B. Toronto ... ... ... ... 24 St. Andrew's Crescent, Cardiff.

Groves, J., M.B., B.A. Lond. ... ... ... ... Carisbrooke.

Guinness, T. A. ... ... ... ... ... ... ... Bampton, Devonshire.

### LIST OF SUBSCRIBERS.

*Hailes, Clements D. G., M.D., C.M. Ed. ... 11 King's Parade, Clifton.
*Haines, A. H. ... ... ... ... ... ... ... ... 8 Cotham Road, Bristol.
*Hale, Edmund T. ... ... ... ... ... ... ... The Grange, Chew Magna.
Handfield-Jones, Montagu, M.D. Lond. ... 24 Montague Square, London, W.
Hardwick, A., M.B. Dur. ... ... ... ... ... New Quay, Cornwall.
Harper, Joseph ... ... ... ... ... ... ... Bear Street, Barnstaple.
Harris, John H. ... ... ... ... ... ... ... Modbury.
*Harrison, A. J., M.B. Lond. ... ... ... ... Guthrie Road, Clifton.
*Harsant, W. H. ... ... ... ... ... ... ... 16 Pembroke Road, Clifton.
Hawkins, Cæsar F. ... ... ... ... ... ... North Petherton.
Haydon, Edgar, M.B., C.M. Glasg. ... ... The Laurels, Newton Abbot.
Hayman, A. G.... ... ... ... ... ... ... ... 1 Pembroke Road, Clifton.
Heane, W. C. ... ... ... ... ... ... ... ... Cinderford.
Hearder, G. J., M.D. St. And.... ... ... ... Joint Counties Asylum, Carmarthen.
*Heaven, John C. ... ... ... ... ... ... ... 2 Queen Square, Bristol.
*Herapath, C. K. C. ... ... ... ... ... ... 11 Brunswick Square, Bristol.
*Hetling, H. E. ... ... ... ... ... ... ... Stapleton Road, Bristol.
Hichens, James Stacey ... ... ... ... ... 17 Green Lane, Redruth.
*Hill, Hedley ... ... ... ... ... ... ... ... General Hospital, Bristol.
Hills, Frederic F. ... ... ... ... ... ... 164 Stapleton Road, Bristol.
Hinton, Joseph ... ... ... ... ... ... ... The Chestnuts, Warminster.
Hoblyn, Francis Parker ... ... ... ... ... 10 Bladud Buildings, Bath.
Hopkins, H. Culliford ... ... ... ... ... 4 Gay Street, Bath.
Hospital, Bristol General (*Sec.*, W. Thwaites).
Hospital, Taunton and Somerset (*House-Surgeon*, W. Burrough Cosens).
Howells, William, M.B., C.M. Glasg. ... Church House, Talgarth, Breconshire.
Howse, William ... ... ... ... ... ... ... 8 London Street, Swindon, Wiltshire.
Howsin, E. Arthur, M.D. St. And. ... ... Stroud.
Hudson, H. R. ... ... ... ... ... ... ... 182 Commercial Road, Newport, Mon.
Huggard, W. R., M.D., M.Ch., M.A. Qu.Univ.I. Davos-Platz, Switzerland.
Hunt, A. D. ... ... ... ... ... ... ... ... Millbrook House, Chagford.
Hutchinson, Jonathan, F.R.S. ... ... ... 15 Cavendish Square, London, W.
Hyatt, Brownlow N.... ... ... ... ... ... Park View, Shepton Mallet.

*Imlay, G. A., M.B., C.M. Aberd. ... ... ... 1 Apsley Villas, Bristol.
Infirmary, Bristol Royal (*Librarian*, Walter J. Hill).
Institution, Liverpool Medical (*Resident Librarian*, William Jones).

Jackson, J. B., M.D., M.Ch.Roy.Univ.I. ... 56 Lemon Street, Truro.
Jacobson, W. H. A., M.B., M.Ch., M.A. Oxon. 66 Great Cumberland Place, London, W.
James, J. Bowen ... ... ... ... ... ... ... Woodlands, Aberaman, Aberdare.
Jessop, Charles Moore ... ... ... ... ... 98 Sutherland Avenue, London, W.
Jones, A. Orlando, M.D., C.M. Aberd. ... Harrogate.
Jones, E. R. ... ... ... ... ... ... ... ... Cilgynlle-fawr, New Quay, Cardiganshire.
Jones, Evan ... ... ... ... ... ... ... ... Ty-Mawr, Aberdare.
Jones, J. Arnalt ... ... ... ... ... ... ... Aberavon.
Jones, W. R., M.D., C.M. Glasg. ... ... ... Senny Bridge, Breconshire.

Kempe, Arthur W. ... ... ... ... ... ... 9 Southernhay, Exeter.
Kendall, Bernard ... ... ... ... ... ... Elm Grove, Tenby

### LIST OF SUBSCRIBERS.

| | |
|---|---|
| Kerr, Elias W., M.B., M. Ch., B.A. Dub. ... | 7 Osborne Terrace, Weymouth. |
| King, Louis J. ... ... ... ... ... ... ... ... | 10 Russel Street, Bath. |
| Kinneir, R. ... ... ... ... ... ... ... ... | The Tower House, Malmesbury. |
| | |
| Lace, F. ... ... ... ... ... ... ... ... ... ... | Royal United Hospital, Bath. |
| Laing, W. A. Gordon, M.D., C.M. Glasg. ... | Boutport Street, Barnstaple. |
| *Lansdown, F. Poole ... ... ... ... ... ... | 19 White Ladies Road, Clifton. |
| *Lawrence, A. E. Aust, M.D., C.M. Aberd. . | 19 Richmond Hill, Clifton. |
| *Lawrence, Henry ... ... ... ... ... ... | 19 Park Row, Bristol. |
| Lawrie, J. Macpherson, M.D., C.M. Glasg. | Greenhill, Weymouth. |
| Leach, J. Comyns, M.D. Dur., B.Sc. Lond. . | Sturminster Newton. |
| Leamon, Michael T. ... ... ... ... ... ... | 1 Bedford Villas, Tavistock. |
| *Lees, E. Leonard, M.B., C.M. Ed. ... ... | Redland Road, Bristol. |
| Lewellyn, John ... ... ... ... ... ... ... | Caerphilly. |
| Library, Bristol Museum and ... ... ... | Queen's Road, Bristol. |
| Library, Cheltenham ... ... ... ... ... | 5 Royal Crescent, Cheltenham. |
| *Lidderdale, James ... ... ... ... ... ... | Bourne End, Maidenhead. |
| Liddon, William, M.B. Lond. ... ... ... ... | Church Square, Taunton. |
| Lilley, G. Herbert, M.D. Brussels ... ... | H.M. Convict Prison, Portland. |
| Limbery, Thomas ... ... ... ... ... ... ... | 184 Commercial Road, Newport, Mon. |
| Lister, Sir Joseph, Bart., F.R.S. ... ... ... | 12 Park Crescent, London, N.W. |
| Lloyd, Walter E. ... ... ... ... ... ... ... | 60 York Road, Bristol. |
| *Logan, Frederic T. B. ... ... ... ... ... | Dean Lane, Bristol. |
| Lowe, T. Pagan ... ... ... ... ... ... ... | 2 Belmont, Bath. |
| Luckham, L. S. ... ... ... ... ... ... ... | 16 Harcourt Terrace, Salisbury. |
| Luffman, W. C. ... ... ... ... ... ... ... | 161 Kennington Road, London, S.E. |
| Lush, William G. Vawdrey, M.D. Lond.... | 12 Frederick Place, Weymouth. |
| *Lyons, A. de Courcy, M.B., C.M. Aberd. ... | Henley Lodge, Yatton, Somersetshire. |
| | |
| MacDonald, P. W., M.D., C.M. Aberd. ... | County Asylum, Dorchester. |
| Maclean, Ewen J., M.B., C.M. Edin. ... ... | Children's Hospital, Bristol. |
| Maclean, J. Campbell, M.B., C.M Ed. ... | Swindon House, Swindon, Wiltshire. |
| Macready, J. ... ... ... ... ... ... ... ... | 51 Queen Anne Street, London, W. |
| *Maddison, W. T., M.D. Lond. ... ... ... ... | 39 Park Street, Bristol |
| Manning, H. J., B.A. Lond. ... ... ... ... | Laverstock House, Salisbury. |
| Marley, Henry F. ... ... ... ... ... ... | The Nook, Padstow, Cornwall. |
| Marsh, Charles J. ... ... ... ... ... ... | Yeovil. |
| Marsh, O. E. Bulwer ... ... ... ... ... | Ventnor House, Newport, Mon. |
| Marshall, Arthur L., M.B., M.A. Cantab. | High Road, London, N.E. |
| *Marshall, Henry, M.D. Ed., F.R.S.E. ... | 28 Caledonia Place, Clifton. |
| *Martin, E. F., M.B. Ed. ... ... ... ... ... | Victoria House, Weston-super-Mare. |
| Martin, Theodore ... ... ... ... ... ... | Temple Cloud. |
| Mason, S. B. ... ... ... ... ... ... ... ... | 12 Park Terrace, Pontypool. |
| Mason, William ... ... ... ... ... ... ... | Sedgemoor Cottage, St. Austell. |
| *Masters, W. Hooper, M.D. St. And. ... ... | 6 Kensington Place, Clifton. |
| *Matthews, Charles E. ... ... ... ... ... | 31 Pembroke Road, Clifton. |
| Mavrojanni, Dr. ... ... ... ... ... ... ... | Corfu, Greece. |
| *Mayor, T. O. ... ... ... ... ... ... ... ... | 40 Apsley Road, Clifton. |
| McNicoll, John ... ... ... ... ... ... ... | High Street, Poole. |
| Meeres, Edward E., M.D. Lond. ... ... ... | 1 St. Andrew's Terrace, Plymouth. |

### LIST OF SUBSCRIBERS.

| | |
|---|---|
| Meredith, John, M.D. Ed. ... ... ... ... | Wellington, Somersetshire. |
| Metford, J. Seymour ... ... ... ... ... | 34 West Mall, Clifton. |
| Michell, George A. ... ... ... ... ... ... ... | Redruth. |
| Milne, John, M.B., C.M. Aberd. ... ... ... | 97 Clarence Road, Bristol. |
| Milward, James, M.D. Brussels ... ... ... | 54 Charles Street, Cardiff. |
| Monckton, William ... ... ... ... ... ... ... | Portishead. |
| Morgan, Frederick ... ... ... ... ... ... ... | Uffculme. |
| Morgan, John ... ... ... ... ... ... ... ... ... | Langport, Somersetshire. |
| Morgan, William, M.D. Aberd. ... ... ... | 16 Edwards Terrace, Cardiff. |
| *Morton, C. A.... ... ... ... ... ... ... ... ... | Zetland Road, Bristol. |
| Moynan, W. Arthur, M.D., M.Ch. Qu. Univ. I. | Wyke House, Isleworth. |
| Munden, Charles ... ... ... ... ... ... ... ... | Ilminster. |
| Murray, William, M.B., C.M. Ed. ... ... | Staple Hill, Gloucestershire. |
| | |
| Napier, T. W. A., M.B., C.M. Aberd. ... ... | Church Street, Egremont, Cheshire. |
| Nash, W. Gifford ... ... ... ... ... ... ... ... | South Devon Hospital, Plymouth. |
| Nevill, W. N., M.B., B.Ch., B.A. Dub. ... | Southville, Bristol. |
| *Newman, Charles ... ... ... ... ... ... ... ... | 19 Portland Square, Bristol. |
| *Newnham, W. H. C., M.B., M.A. Cantab. ... | General Hospital, Bristol. |
| Newstead, James ... ... ... ... ... ... ... ... | 9 York Place, Clifton. |
| Newton, Charles John ... ... ... ... ... ... | Oriel Lodge, Cheltenham. |
| Nicholson, T. D., M.D., C.M. Ed. ... ... | 2 White Ladies Road, Clifton. |
| Norman, J. H. ... ... ... ... ... ... ... ... ... | Goodleigh, Winkleigh, Devonshire. |
| *Norton, J. A., M.D., C.M. Aberd ... ... ... | 65 Redcliff Hill, Bristol. |
| | |
| Ollerhead, T. J. ... ... ... ... ... ... ... ... | Hillbury, Minehead, Somersetshire. |
| *Ormerod, Henry ... ... ... ... ... ... ... ... | Westbury-on-Trym. |
| | |
| Padley, George ... ... ... ... ... ... ... ... | 2 Northampton Terrace, Swansea. |
| Page, G. Shepley ... ... ... ... ... ... ... ... | 78 Old Market Street, Bristol. |
| Pain, Tertius D'Oyley ... ... ... ... ... ... | Bembridge, Isle of Wight. |
| Paine, William Henry, M.D. Aberd. ... ... | Stroud. |
| Palmer, F. Craddock, M.D. Brussels ... ... | The Corderries, Chalford. |
| Papillon, J. W. ... ... ... ... ... ... ... ... | The Firs, Brent Knoll. |
| *Parker, George, M.D., M.A. Cantab. ... ... | 14 Pembroke Road, Clifton. |
| Parry, J. H. ... ... ... ... ... ... ... ... ... | 99 Ashley Road, Bristol. |
| *Parson, T. Cooke ... ... ... ... ... ... ... ... | 21 White Ladies Road, Bristol. |
| Parsons, Francis J. C. ... ... ... ... ... ... | King Square, Bridgwater. |
| Parsons, Frederick ... ... ... ... ... ... ... | Common Hill, Frome. |
| Paterson, Donald Rose, M.D., C.M. Ed.... | 7 Newport Road, Cardiff. |
| Pauli, G. C. P. ... ... ... ... ... ... ... ... | 240 York Road, Bristol. |
| Paull, Josiah ... ... ... ... ... ... ... ... ... | Camborne. |
| Pearse, W. ... ... ... ... ... ... ... ... ... ... | St. Tudy, Bodmin. |
| *Penny, F. ... ... ... ... ... ... ... ... ... ... | 42 Caledonia Place, Clifton. |
| *Penny, W. J. ... ... ... ... ... ... ... ... ... | 6 Chandos Street, London, W. |
| Pentland, Young J. ... ... ... ... ... ... ... | 11 Teviot Place, Edinburgh. |
| Perkins, J. H., M.D. New York ... ... ... | 14 Victoria Square, Clifton. |
| Peskett, A. W. C., M.B., B.C., M.A. Cantab. | Burnham, Somersetshire. |
| Phelps, W. Harford G., M.D. Aberd. ... | Beachfield, Weston-super-Mare. |

### LIST OF SUBSCRIBERS.

| | |
|---|---|
| PHILLIPS, E. ENGLAND ... ... ... ... ... ... | Broadwater House, Southend. |
| *PICKERING, C. F. ... ... ... ... ... ... ... | 12 Berkeley Square, Bristol. |
| PIPPETTE, WALTER... ... ... ... ... ... ... | 90 Brooke Road, London, N. |
| PIZEY, G. F. P.... ... ... ... ... ... ... ... | Rydal Villa, Clevedon. |
| PLAISTER, W. H. ... ... ... ... ... ... ... | High Road, Tottenham. |
| POLLARD, REGINALD, M.B. Dur. ... ... ... | Southlands, Torquay. |
| *POUNTNEY, W. E., M.D., C.M. Ed. ... ... | 33a White Ladies Road, Bristol. |
| POWELL, R. DOUGLAS, M.D. Lond.... ... ... | 62 Wimpole Street, London, W. |
| POWELL, WILLIAM, M.B. Lond. ... ... ... | Hill Garden, Torquay. |
| PRICE, WILLIAM, M.B. Lond. ... ... ... ... | 40 Park Place, Cardiff. |
| *PRICHARD, ARTHUR W.... ... ... ... ... ... | Gordon Road, Clifton. |
| *PRICHARD, AUGUSTIN, M.D. Berlin... ... ... | 4 Chesterfield Place, Clifton. |
| *PRICHARD, J. E., M.B., B.A. Oxon. ... ... | 243 Cheltenham Road, Bristol. |
| PRICHARD, R., M.D., C.M. Glasg. ... ... ... | 14 Windsor Place, Cardiff. |
| PRIDEAUX, T. ENGLEDUE P. ... ... ... ... | Wellington, Somersetshire. |
| *PRING, ARTHUR, B.A. Cantab. ... ... ... ... | 22 Meridian Place, Clifton. |
| PROWSE, ALBERT P. ... ... ... ... ... ... | Yennadon, Horrabridge, Devonshire. |
| *PROWSE, ARTHUR B., M.D. Lond. ... ... ... | 5 Lansdown Place, Clifton. |

| | |
|---|---|
| RANDOLPH, CHARLES ... ... ... ... ... ... | St. Michael's, Milverton, Somersetshire. |
| RATTRAY J. M., M.B., C.M., M.A. Aberd. ... | Frome. |
| RAWLINGS, JOHN ADAMS ... ... ... ... ... | 4 Northampton Terrace, Swansea. |
| REDWOOD, T. HALL, M.D., C.M., M.A. Dur.... | The Lawn, Rhymney, Monmouthshire. |
| RENDALL, JOHN... ... ... ... ... ... ... ... | Forestside, Lymington. |
| RIDDELL, ANDREW W. ... ... ... ... ... ... | Westbury-on-Trym. |
| ROBATHAN, G. B. ... ... ... ... ... ... ... | The Grove, Risca. |
| ROLSTON, G. T. ... ... ... ... ... ... ... | 8 Osborne Villas, Stoke, Devonport. |
| *ROSS, R. A. ... ... ... ... ... ... ... ... | Lodway Villa, Pill. |
| *ROSSITER, GEORGE F., M.B. Lond. ... ... | Cairo Lodge, Weston-super-Mare. |
| *ROUÉ, W. BARRETT, M.D., M.S. Dur. ... ... | 2 Buckingham Place, Clifton. |
| *ROXBURGH, ROBERT, M.B. Ed. ... ... ... ... | 1 Victoria Buildings, Weston-super-Mare. |
| ROYLE, JOHN FREDERICK S., M.B., C.M. Aberd. | 30 Foregate Street, Worcester. |
| *RUDGE, C. KING ... ... ... ... ... ... ... | 145 White Ladies Road, Bristol. |
| *RUDGE, H. T. ... ... ... ... ... ... ... ... | 5 Colston Parade, Bristol. |
| RUNDLE, EDMUND ... ... ... ... ... ... ... | Royal Cornwall Infirmary, Truro. |
| RUSSELL, M. W. H. ... ... ... ... ... ... | 6 Whitehall Yard, London, S.W. |

| | |
|---|---|
| SCIMONELLI, SIGra. CRUCE ... ... ... ... | 5 Salita dei Cattivi, Via Butera, Palermo. |
| SCOTT, JAMES, M.B., C.M. Ed.... ... ... ... | 24 Eastgate Street, Stafford. |
| SCOTT, RICHARD J. H. ... ... ... ... ... ... | 13 Bladud Buildings, Bath. |
| SEARLE, GEORGE C. ... ... ... ... ... ... | Brixham, Devonshire. |
| SEARLE, RICHARD BURFORD ... ... ... ... | St. Just. |
| *SEMPLE, H. F.... ... ... ... ... ... ... ... | 3 Fairview Villas, Redland, Bristol. |
| SEWARD, W. J., M.B. Lond. ... ... ... ... | Asylum, Colney Hatch, London, N. |
| SEWART, J. H. ... ... ... ... ... ... ... ... | Lostwithiel. |
| SHAPTER, LEWIS, M.D., B.A. Cantab. ... ... | The Barnfield, Exeter. |
| SHARPE, J. HENRY ... ... ... ... ... ... ... | Greenwood, Huntspill. |
| *SHAW, J. E., M.B., C.M. Ed. ... ... ... ... | 11 Lansdown Place, Clifton. |
| SHEEN, A., M.D. St. And. ... ... ... ... ... | 23 Newport Road, Cardiff. |
| *SHEPPARD, E. J. ... ... ... ... ... ... ... | 16 Park [illegible]ace, Clifton. |

### LIST OF SUBSCRIBERS.

| | |
|---|---|
| *Shorland, Walter E. ... ... ... ... ... | 189 Cheltenham Road, Bristol. |
| Shortridge, Thomas Wood, M.D. Brussels | Honiton. |
| Sincock, J. Bain ... ... ... ... ... ... ... | Friarn Street, Bridgwater. |
| Skeate, Edwin ... ... ... ... ... ... ... | 16 Paragon, Bath. |
| *Skelton, Henry, M.D. St. And. ... ... ... | Downend, Gloucestershire. |
| *Skerritt, E. Markham, M.D., B.S., B.A. Lond. | Richmond Hill, Clifton. |
| *Skinner, Stephen, M.B., C.M. Aberd. ... | Ferndale, Clevedon. |
| Smith, Frederick A. A., M.D., C.M. Glasg. | Portland House, Cheltenham. |
| *Smith, G. Munro ... ... ... ... ... ... ... | 27 All Saints' Road, Clifton. |
| *Smith, J. Greig, M.B., C.M., M.A. Ab., F.R.S.E. | 16 Victoria Square, Clifton. |
| Smith, J. Sidney, M.D. St. And. ... ... ... | Tiverton. |
| *Smith, R. Shingleton, M.D., B.Sc. Lond. | Clifton Park, Clifton. |
| Smith, Samuel ... ... ... ... ... ... ... | 7 Kingsdown Parade, Bristol. |
| Smyth, Spencer T., M.D. Aberd. ... ... ... | Lansdowne Road, Bournemouth. |
| Society, Cardiff Medical (*Hon. Sec.*, Dr. D. R. Paterson). | |
| Society, Royal Medical and Chirurgical | 53 Berners Street, London, W. |
| Somer, James ... ... ... ... ... ... ... ... | Broadclyst. |
| *Spencer, W. H., M.D., M.A. Cantab. ... ... | 11 Warrior Square, St. Leonard's-on-Sea. |
| Spender, John K., M.D. Lond. ... ... ... | 17 Circus, Bath. |
| Stamp, W. D. ... ... ... ... ... ... ... ... | Ridgeway House, Plympton. |
| Stansfeld, G. M. ... ... ... ... ... ... ... | 19 Victoria Square, Clifton. |
| Statham, R. Whiteside ... ... ... ... ... | Cheddar, Somersetshire |
| Steel, Arthur R.... ... ... ... ... ... ... | Fern Villa, Belle Vue, Wakefield. |
| *Steele, Charles, M.D. Dur. ... ... ... ... | 17 Richmond Hill, Clifton. |
| Steele-Perkins, E. B. ... ... ... ... ... | 20 Sidwell Street, Exeter. |
| Stephens, Lockhart E. W. ... ... ... ... | Emsworth. |
| *Stevens, W. H. ... ... ... ... ... ... ... | 221 Cheltenham Road, Bristol. |
| Stewart, James, B.A. Qu. Univ. I. ... ... | Dunmurry, Stoke Bishop. |
| Stoddart, F. Wallis ... ... ... ... ... ... | 1 Park Street, Bristol. |
| Storry, Frederick W. ... ... ... ... ... | Lansdown, Stroud. |
| *Swain, James, M.D., B.S. Lond. ... ... ... | Royal Infirmary, Bristol. |
| *Swayne, J. G., M.D. Lond. ... ... ... ... | 74 Pembroke Road, Clifton. |
| *Swayne, S. H.... ... ... ... ... ... ... ... | 129 Pembroke Road, Clifton. |
| *Swayne, W. Carless, M.B. Lond.... ... ... | St. Paul's Road, Clifton. |
| Sydenham, George F. ... ... ... ... ... | Dulverton, Somersetshire. |
| Syrée, A. Hugh ... ... ... ... ... ... ... | County Asylum, Devizes. |

| | |
|---|---|
| Tait, Lawson ... ... ... ... ... ... ... ... | 7 The Crescent, Birmingham |
| Taylor, C. Bell, M.D. Edin. ... ... ... ... | 9 Park Row, Nottingham. |
| *Taylor, James... ... ... ... ... ... ... ... | 36 Alma Road, Clifton. |
| Taylor, Thomas Henry ... ... ... ... ... | Thornbury, Gloucestershire. |
| Thomas, A. Garrod, M.D., C.M. Ed. ... ... | Clytha Park, Newport, Mon. |
| Thomas, Jabez ... ... ... ... ... ... ... | Ty Cerrig, Swansea. |
| Thomas, John T. ... ... ... ... ... ... ... | Chepstow Road, Newport, Mon. |
| Thomas, R. W. ... ... ... ... ... ... ... | Freemantle Square, Bristol. |
| Thompson, J. Tatham, M.B., C.M. Ed. ... | 23 Charles Street, Cardiff. |
| Thomson, Eustace B., M.D. C.M. Glasg.... | Albany Place, Plymouth. |
| *Tivy, W. J. ... ... ... ... ... ... ... ... | 8 Lansdown Place, Clifton. |
| *Tomkins, Harding H.... ... ... ... ... ... | 8 Auckland Terrace, Ramsey, Isle of Man. |
| Torbock, W. H., M.D. Pennsylvania ... ... | Polruan, Cornwall. |
| Tosswill, Louis H., M.B., B.A. Cantab. ... | 28 West Southernhay, Exeter. |

### LIST OF SUBSCRIBERS.

| | |
|---|---|
| Trotter, Leslie B., M.D., C.M. Ed. ... ... | Coleford, Gloucestershire. |
| Twining, A. H. ... ... ... ... ... ... ... | The Knoll, Salcombe. |
| Tyley, R. P., M.D. St. And. ... ... ... ... | Wedmore. |
| | |
| Vachell, C. T., M.D. Lond. ... ... ... ... | 38 Charles Street, Cardiff. |
| Vachell, Herbert R., M.D., C.M. Aberd.... | 15 Newport Road, Cardiff. |
| Vernon, Edward ... ... ... ... ... ... ... | Kingston, Yeovil. |
| | |
| Wade, A. Law, M.D., B.A. Dub. ... ... ... | County Asylum, Wells, Somersetshire. |
| *Waldo, Henry, M.D., C.M. Aberd. ... ... | 19 Pembroke Road, Clifton. |
| Wallace, Rev. C. Hill, M.A. Oxon. ... ... | 3 Harley Place, Clifton. |
| Walter, William Henry, M.D. Brussels ... | Knap Villa, South Petherton. |
| Ward, J. L. W. ... ... ... ... ... ... ... | Victoria Street, Merthyr Tydvil. |
| *Wathen, J. Hancocke ... ... ... ... ... | 16 York Place, Clifton. |
| Watson, J. Adam ... ... ... ... ... ... ... | 39 Dennington Park, London, N.W. |
| Watters, George T. B., M.D., C.M. Ed. ... | Stonehouse, Gloucestershire. |
| Waugh, Alexander ... ... ... ... ... ... | Midsomer Norton. |
| *Weatherly, Lionel A., M.D., C.M. Aberd. | Bailbrook House, Bath. |
| Webber, William W.... ... ... ... ... ... | Crewkerne. |
| Webster, Norman P. ... ... ... ... ... ... | George Place, Guernsey. |
| *Webster, Thomas ... ... ... ... ... ... | Redland Hill, Bristol. |
| *Wedmore, C. Ernest, M.B., M.A. Cantab. | 11 Richmond Hill, Clifton. |
| Whishaw, Reginald R., M.B., B.C., B.A. Cantab. | St. Paul's Road, Clifton. |
| Whitfeld, W. Clarke ... ... ... ... ... ... | 5 St. Ethelbert Street, Hereford. |
| Whyte, Andrew, M.D., C.M. Aberd. ... ... | Honddû House, Brecon. |
| Wickham, W. ... ... ... ... ... ... ... ... | Hill House, Tetbury. |
| Wicksteed, Francis W. S. ... ... ... ... | The Villa Rosa, Weston-super-Mare |
| Wigan, Charles A., M.D. Dur. ... ... ... | Portishead. |
| Wigmore, J., M.D. Qu. Univ. I.... ... ... ... | Twerton-on-Avon. |
| *Wilding, James, M.D. Dur. ... ... ... ... | Sydenham Road, Bristol. |
| Willcox, R. Lewis ... ... ... ... ... ... | Warminster. |
| Willett, George G. D. ... ... ... ... ... | Keynsham. |
| Williams, Charles ... ... ... ... ... ... | Port Isaac. |
| Williams, D. J. ... ... ... ... ... ... ... | Greenfield House, Llanelly. |
| Williams, H. Egerton ... ... ... ... ... | The Mount, Newport, Mon. |
| Williams, Lionel H. ... ... ... ... ... ... | The County Hospital, York. |
| *Williams, P. Watson, M.B. Lond. ... ... | 14 Westbourne Place, Clifton. |
| Williams, Peter ... ... ... ... ... ... ... | Ferryside, Carmarthenshire. |
| Williams, William Henry, M.D. Lond. ... | Sherborne. |
| Wilson, Claude, M.D., C.M. Ed. ... ... ... | Church Road, Tunbridge Wells. |
| *Windsor-Aubrey, H. W. ... ... ... ... ... | Coronation Road, Bristol. |
| Winterbotham, L. ... ... ... ... ... ... | Bays Hill, Cheltenham. |
| Woodd, H. T. ... ... ... ... ... ... ... ... | Kelly Villa, Calstock. |
| | |
| Yelf, Leonard K., M.D. St. And. ... ... ... | Moreton-in-Marsh. |

### Bristol Medico-Chirurgical Journal.

*Cases for binding, price 7d. each, may be had of the Publisher,* J. W. ARROWSMITH, *Quay Street, Bristol.*

### Exchange-List.

### AFRICA.

### CAPE COLONY.

*Weekly.*

*The South African Medical Journal.* East London: W. Darley-Hartley.

### AMERICA.

### ARGENTINE REPUBLIC.

*Monthly.*

*Anales del Circulo Médico Argentino.* Buenos Ayres: M. Biedma.

### CANADA.

*Monthly.*

*The Montreal Medical Journal.* Montreal: Gazette Printing Company.

### PERU.

*Monthly.*

*La Crónica Médica.* Lima: Carlos Prince.

### UNITED STATES.

*Weekly.*

*The Cincinnati Lancet-Clinic.* Cincinnati: 281 West 7th Street.

*The Journal of the American Medical Association.* Chicago: 65 Randolph Street.

*The Medical and Surgical Reporter.* Philadelphia: 115 South Seventh Street.

*The Medical News.* Philadelphia: Lea Brothers & Co.

*The Medical Record.* New York: William Wood & Co.

*The New York Medical Journal.* New York: D. Appleton & Co.

*Fortnightly.*

*The American Practitioner & News.* Louisville: John P. Morton and Company.

*Twice a month.*

*The Medical Age.* Detroit: George S. Davis.

*Monthly.*

*Index Medicus.* Detroit: George S. Davis.

*Journal of Cutaneous and Genito-Urinary Diseases.* New York: D. Appleton & Co.

*The Abstract and Index.* Weston: H. H. Howe.

*The American Journal of Ophthalmology.* St. Louis: J. H. Chambers & Co.

*Monthly.*

*The American Journal of the Medical Sciences.* Philadelphia: Lea Brothers & Co.

*The Medical Analectic and Epitome.* New York: G. P. Putnam's Sons.

*The Obstetric Gazette.* Cincinnati: 281 West 7th Street.

*The Saint Louis Medical and Surgical Journal.* Saint Louis: 2622 Washington Avenue.

*The Therapeutic Gazette.* Detroit: George S. Davis.

*Yearly.*

*Annual of the Universal Medical Sciences.* Philadelphia: F. A. Davis.

*Transactions of the American Surgical Association.* Philadelphia: P. Blakiston, Son & Co.

*Occasionally.*

*Transactions of the New York Academy of Medicine.* New York: 12 West 31st Street.

### ASIA.

### JAPAN.

*Monthly.*

*The Sei-I-Kwai Medical Journal.* Tokio: Z. P. Maruya & Co.

### AUSTRALASIA.

### AUSTRALIA.

*Monthly.*

*The Australasian Medical Gazette.* Sydney L. Bruck.

*The Australian Medical Journal.* Melbourne: Stillwell & Co.

### EUROPE.

### AUSTRIA-HUNGARY.

*Weekly.*

*Orvosi hetilap.* Buda-Pesth: Dr. Markusovszky.

### BELGIUM.

*Monthly.*

*Bulletin de l'Académie royale de médecine de Belgique.* Brussels: F. Hayez.

### BRITISH ISLES.

*Weekly.*

*British Medical Journal.* London: 429 Strand.

*The Hospital.* London: 38 Old Jewry.

*The Lancet.* London: 423 Strand

*Monthly.*

*Annals of Surgery.* London: Baillière, Tindall & Cox.

*The Birmingham Medical Review.* Birmingham: Cornish Bros.

*The Journal of Laryngology and Rhinology.* London: R. Anderson & Co.

*The London Medical Recorder.* London: W. H. Allen & Co.

*The Medical Chronicle.* Manchester: John Heywood.

*The Practitioner.* London: Macmillan & Co.

*Edinburgh Medical Journal.* Edinburgh: Oliver and Boyd.

*The Glasgow Medical Journal.* Glasgow: Alex. Macdougall.

*The Dublin Journal of Medical Science.* Dublin: Fannin & Co.

*Quarterly.*

*The Asclepiad.* London: Longmans, Green, & Co.

*The British Gynæcological Journal.* London: John Bale & Sons.

*Half-yearly.*

*The Liverpool Medico-Chirurgical Journal.* Liverpool: Medical Institution.

*The Retrospect of Medicine.* London: Simpkin, Marshall, & Co.

*Yearly.*

*The Directory of Second-hand Booksellers.* Rochdale: James Clegg.

*The Medical Annual.* Bristol: John Wright & Co.

*The Year-Book of Treatment.* London: Cassell & Company, Limited.

*Transactions of the Academy of Medicine in Ireland.* Dublin: Fannin & Co.

*Year-Book of Scientific and Learned Societies.* London: C. Griffin & Co.

DENMARK.

*Weekly.*

*Hospitals-Tidende.* Copenhagen: Jacob Lund.

FRANCE.

*Three times a week.*

*Gazette des hôpitaux.* Paris: 4 Rue de l'Odéon.

*L'Union médicale.* Paris: 11 Rue de la Grange-Batelière.

*Weekly.*

*Bulletin de l'Académie de médecine.* Paris: G. Masson.

*Le Progrès médical.* Paris: 14 Rue des Carmes.

*Twice a month.*

*Gazette de Gynécologie* Paris: O. Doin.

*Monthly.*

*Revue mensuelle de Laryngologie, d'Otologie et de Rhinologie.* Paris: 8 Place de l'Odéon.

GERMANY.

*Twice a week.*

*Deutsche Medizinal-Zeitung.* Berlin: Eugen Grosser.

*Weekly.*

*Medicinische Bibliographie.* Leipzig: Breitkopf & Härtel.

HOLLAND.

*Weekly.*

*Nederlandsch Tijdschrift voor Geneeskunde.* Amsterdam: F. van Rossen.

ITALY.

*Daily.*

*La Riforma Medica.* Naples: 48 Sette Dolori a Toledo.

*Monthly.*

*Lo Sperimentale.* Florence: 35 Via degli Alfani.

NORWAY.

*Monthly.*

*Norsk Magazin for Lægevidenskaben.* Christiania: Th. Steens.

PORTUGAL.

*Weekly.*

*A Medicina Contemporanea.* Lisbon: José Antonio Rodrigues & Ca.

ROUMANIA.

*Twice a Month.*

*Spitalul.* Bucharest: Stefan Mihalescu.

RUSSIA.

*Weekly.*

*Medycyna.* Warsaw: 80 Allée de Jérusalem

*Vrach.* St. Petersburg: C. Ricker.

SPAIN.

*Weekly.*

*El Siglo Médico.* Madrid: Magdalena, 36, 2°

SWEDEN.

*Quarterly.*

*Nordiskt Medicinskt Arkiv.* Stockholm: P. A. Norstedt & Söner.

SWITZERLAND.

*Monthly.*

*Illustrirte Monatsschrift der ärztlichen Polytechnik und Centralblatt der orthopädischen Chirurgie.* Berne: Schmid, Francke & Co.

### *PUBLICATIONS RECEIVED.*

From 5 Avenue de l'Opéra, Paris:
*Annales d'Orthopédie et de Chirurgie pratiques.* Nos. 1, 2. 1890.
From 3 Rue Christine, Paris:
*Revue chirurgicale.* Nos. 1, 2, 4. 1889. Nos. 2, 4, 5. 1890.
From Gress & Sexton, Atlanta:
*The Southern Medical Record.* January, 1890.
From William Wood & Co., New York:
*Concealed Pregnancy.* By Albert Vander Veer, M.D. 1889. Reprint.
From 95 William Street, New York:
*The International Journal of Surgery.* December, 1889.
From F. A. Davis, Philadelphia:
*Materia Medica, Pharmacology, and Therapeutics.* By John V. Shoemaker, A.M., M.D., and John Aulde, M.D. In two volumes. Vol. I. 1889.
*Essay on Medical Pneumatology.* By J. N. Demarquay. Translated, with Notes, Additions, and Omissions, by S. S. Wallian, A.M., M.D. 1889.
*Spinal Concussion.* By S. V. Clevenger, M.D. 1889.
*The Medical Bulletin.* January, 1890.
From Wm. J. Dornan, Philadelphia:
*Uterine Cancer.* By John Byrne, M.D. 1889. Reprint.
From The Medical Press Company, Limited, Philadelphia:
*The Dietetic Gazette.* December, 1889.
From Ev. E. Carreras, St. Louis:
*The Alienist and Neurologist.* January, 1890.
From W. L. Klein, St. Paul, Minnesota:
*Northwestern Lancet.* October 15th, 1889.
From John Wright & Co., Bristol:
*Children's Deformities.* By Walter Pye. [1889.]
From The Liberal Union of Ireland, Dublin:
*The Plan of Campaign: The Ponsonby Estate.* February, 1890.
From Young J. Pentland, Edinburgh:
*Studies in Clinical Medicine.* By Byrom Bramwell, M.D. Nos. 13—19. 1889-90.
*Reports from the Laboratory of the Royal College of Physicians, Edinburgh.* Edited by J. Batty Tuke, M.D., and G. Sims Woodhead, M.D. Vol. II. 1890.
From Baillière, Tindall, & Cox, London:
*Bacteria in Animal Tissues.* By Dr. H. Kühne. 1890.
From T. B. Burrow, London:
*The News.* January 17th, 1890.
From Cassell & Company, Limited, London:
*Food in Health and Disease.* By I. Burney Yeo, M.D. 1889.
*The Pulse.* By W. H. Broadbent, M.D. 1890.
From Charles Griffin and Company, London:
*A Manual of Nursing.* By Laurence Humphry, M.A., M.B. 1889.
From H. K. Lewis, London:
*The British Journal of Dermatology.* December, 1889. January—March, 1890.
*Insomnia and its Therapeutics.* By A. W. Macfarlane, M.D. 1890.
*History and Pathology of Vaccination.* By Edgar M. Crookshank, M.B. Two volumes. 1889.
*The Middlesex Hospital: Reports of the Medical, Surgical, and Pathological Registrars for the Year* 1888. 1889.
*Leprosy.* By Robson Roose, M.D. 1890.
*Influenza.* By J. Ernest Viney, M.A., M.D. 1890.
*Asthma.* By E. Schmiegelow, M.D. 1890.
*The Natural History of Specific Diseases.* By E. F. Willoughby, M.D. 1890.
*Wanderings in Search of Health.* By H. Coupland Taylor, M.D. 1890.
From Kegan Paul, Trench & Co., London:
*Egypt as a Winter Resort.* By F. M. Sandwith. 1889.
From John Morgan Richards, London:
*Medical Reprints.* No. 1. February 15th, 1890.
From The Illustrated Medical News Publishing Co., Limited, London:
*The Illustrated Medical News.* January 18th, 1890.

